# Elucidation of the Rotavirus NSP4-Caveolin-1 and -Cholesterol Interactions Using Synthetic Peptides

**DOI:** 10.1155/2012/575180

**Published:** 2012-03-01

**Authors:** Megan E. Schroeder, Heather A. Hostetler, Friedhelm Schroeder, Judith M. Ball

**Affiliations:** ^1^Department of Veterinary Pathobiology, Texas A&M University, TVMC, College Station, TX 77843-4467, USA; ^2^Molecular Diagnostics Texas Veterinary Medical Diagnostic Laboratory, College Station, TX 77843, USA; ^3^Department of Pharmacology and Physiology, Texas A&M University, TVMC, College Station, TX 77843-4467, USA; ^4^Department of Biochemistry and Molecular Biology, Boonshoft School of Medicine, Diggs 056, 3640 Colonel Glenn Hwy, Dayton, OH 45435, USA

## Abstract

Rotavirus (RV) NSP4, the first described viral enterotoxin, is a multifunctional glycoprotein that contributes to viral pathogenesis, morphogenesis, and replication. NSP4 binds both termini of caveolin-1 and is isolated from caveolae fractions that are rich in anionic phospholipids and cholesterol. These interactions indicate that cholesterol/caveolin-1 plays a role in NSP4 transport to the cell surface, which is essential to its enterotoxic activity. Synthetic peptides were utilized to identify target(s) of intervention by exploring the NSP4-caveolin-1 and -cholesterol interactions. NSP4_112–140_ that overlaps the caveolin-1 binding domain and a cholesterol recognition amino acid consensus (CRAC) motif and both termini of caveolin-1 (N-caveolin-1_2–20_,  _19–40_ and C-caveolin-1_161–180_) were synthesized. Direct fluorescence-binding assays were employed to determine binding affinities of the NSP4-caveolin-1 peptides and cholesterol. Intracellular cholesterol alteration revealed a redistribution of NSP4 and disintegration of viroplasms. These data further imply interruption of NSP4_112–140_-N-caveolin-1_19–40_ and cholesterol interactions may block NSP4 intracellular transport, hence enterotoxicity.

## 1. Introduction

As the leading cause of gastroenteritis in young children under the age of five, rotavirus (RV) infections annually are responsible for approximately 600,000 deaths worldwide [1, 2]. During infection, the RV nonstructural protein 4 (NSP4) functions as a viral enterotoxin by binding an extracellular receptor and activating a signal transduction pathway which increases intracellular calcium ([Ca^2+^]_i_) levels through the release of ER calcium stores [3, 4]. This increase in [Ca^2+^]_i_ induces secretory chloride currents which result in diarrhea, but only when initiated from the exofacial leaflet of the plasma membrane (PM) [3]. The initiated increases in intracellular, calcium levels fail to induce the chloride secretory response [4–6].

Traditionally defined as an ER glycoprotein, NSP4 contains a single transmembrane domain that serves to anchor the protein into the ER membrane, such that a short N-terminal domain (amino acids [aa] 1–24) remains within the lumen of the ER while the longer C-terminus (aa 45–175) extends into the cytoplasm [7]. Interactions with numerous viral and cellular proteins occur within the extended C-terminal tail of NSP4 [8–12]. Also contained within the C-terminal cytoplasmic tail is an amphipathic *α*-helix (AAH), coiled-coil domain (aa 95–137) [13, 14]. Cross-linking and crystallographic experiments reveal that this region of NSP4 primarily oligomerizes into dimers and tetramers and contains a cation-binding site [13–15]. Residing within the amphipathic *α*-helix, coiled-coil domain is the enterotoxic peptide region (aa 114–135) as well as the caveolin-1 (cav-1) binding domain [16] and a putative cholesterol recognition amino acid consensus (CRAC) sequence [17].

Many cholesterol binding proteins contain a CRAC sequence characterized by—L/V-(X)_(1-5)_-Y-(X)_(1-5)_-R/K-, where (X)_(1-5)_ represents between one and five unspecified residues [17]. Numerous proteins involved in cholesterol transport contain this sequence, including cav-1 [18]. However, the presence of such a sequence does not assure an interaction with cholesterol, as this sequence is highly variable and likely is not the only requirement for cholesterol binding [19]. Yet a number of viral proteins bind cholesterol, including the HIV-1 gp41 transmembrane glycoprotein [17, 20], the influenza M2 protein [21], and the F1 subunit of the fusion protein of Sendai virus [22].

There is compelling evidence that the enterotoxic peptide, NSP4_114-135_, and the full-length NSP4 protein interact with cav-1. *In vivo* laser scanning confocal microscopy (LSCM) colocalizations, fluorescent energy transfer (FRET) analyses, and co-immunoprecipitation data from our laboratory verify that NSP4 binds cav-1, the main constituent protein of caveolae, at multiple sites within the cell, including the PM [23]. Yeast two-hybrid and *in vitro* binding assays confirm an interaction between NSP4 and cav-1 and map the cav-1 binding domain to NSP4_114-135_ [23]. Using deletion and site-directed mutagenesis, the binding site delineated to three hydrophobic residues in the enterotoxic region indicating that NSP4 and cav-1 associate via a hydrophobic interaction (unpublished). Additional studies reveal that the cav-1 binding site for NSP4 maps, to both the N- and C-termini of cav-1, residues 2–31 and 161–178, respectively [24].

Cav-1 is an intracellular, 21 kD protein bound by the inner leaflet of the PM in a hairpin-like structure such that both the N- and C-termini are oriented towards the cytoplasm [23, 25]. Numerous signaling molecules, such as those involved in calcium signaling, are localized to caveolae and their function appears to be dependent on interaction with cav-1 [16, 26, 27]. Cav-1 also binds cholesterol, which has been shown to be vital to caveolae biogenesis and the transport of cholesterol to caveolae at the PM [28–31].

Purified NSP4 and NSP4_114-135_ peptides interact with caveolae-like model membranes, that is, those that have a high radius of curvature and are rich in anionic phospholipids and cholesterol [32, 33]. Upon interaction with model membranes, both the protein and the peptide undergo a change in secondary structure characterized by an increase in *α*-helix formation. Additional secondary structure studies of other NSP4 peptides that overlap the enterotoxic peptide and cav-1 binding domain confirm that this region is important for membrane interactions. When caveolae are isolated from enriched PM fractions of RV-infected MDCK cells, the full-length, fully-glycosylated NSP4 is present in the isolated caveolae fractions verifying transport of NSP4 to the PM and caveolae [34]. It is possible that the transport of NSP4 to caveolae occurs through an interaction with cav-1 and cholesterol, although additional studies are needed to for verification. Our studies reveal the exposure of the NSP4 C-terminus and the enterotoxic region, but not the N-terminus, on the outer PM leaflet and subsequent release into culture media at a time when the membrane is intact as verified by a lack of exposure of cav-1 and other intracellular markers [34–36].

In this paper, we have extended these studies using synthetic peptides to address the following questions. (i) Does NSP4 interact with both termini of cav-1 with equal affinity? (ii) Do the two termini of cav-1 interact with one another and thus facilitate binding of NSP4 to both termini? (iii) Is there a structural change when NSP4 and cav-1 peptides interact? (iv) Do the overlapping NSP4-cav-1 binding, CRAC motif, and enterotoxin regions interact with cholesterol? (v) Does alteration(s) of NSP4-cholesterol binding have a biological effect? To answer these questions, we employed peptide-peptide and -cholesterol direct fluorescence-binding assays to ascertain the interaction and determine the binding affinities (*K*_*d*_). We also employed circular dichroism (CD) to investigate secondary structural changes upon interaction of the peptides in the absence or presence of model membranes.

## 2. Materials and Methods

### 2.1. Materials, Antibodies and Cells

Trifluoroacetic acid (TFA), 1-hydroxy-benzotriazole (HOBt), and O-Benzotriazole-N,N,N′,N′-tetramethyluronium-hexafluoro-phosphate (HBTU) were purchased from American Bioanalytical (Natick, MA). N,N-diisopropylethylamine (DIPEA) and N,N′-diisopropylcarbodiimide (DIPCIDI) were purchased from CreoSalus (Louisville, KY). Thioanisole, ethanedithiol, and anisole were purchased from EMD chemicals (Gibbstown, NJ). Cholesterol, Sephadex G-25, and trifluoroethanol (TFE) were purchased from Sigma (St. Louis, MO). Phospholipids DOPS (1,2-dioleoyl-*sn*-glycero-3-[phosphor-L-serine]) and POPC (1-palmitoyl-2-oleoyl-*sn*-glycero-3-phosphocholine) were purchased from Avanti Polar Lipids (Alabaster, AL).

 Antibodies specific to the NSP4 peptide aa 150–175 (NSP4_150-175_; deduced from the simian rotavirus SA11 NSP4 sequence) were generated in rabbits by immunizing with peptide cross-linked to keyhole limpet hemocyanin as previously described [23]. Anti-NSP4_150-175_ was affinity purified against the inoculating peptide linked to preactivated cyanogen bromide Sepharose 4B beads according to the manufacturer (Amersham Pharmacia Biotech, Piscataway, NJ) [37]. Guinea pig anti-NSP5, a viroplasm (virus factory in the cell) marker, was a gift from Dr. Oscar Burrone (International Center for Genetic Engineering and Biotechnology, Trieste, Italy). F(ab′)_2_ fragments of goat anti-guinea pig IgG-Cy3 and goat anti-rabbit IgG-Cy2 were purchased from Jackson Immuno Research (West Grove, PA).

 HT29.f8 cells are a spontaneously polarizing cell line that was cloned from the human adenocarcinoma HT29 intestinal cell line [38]. Cells were maintained in Dulbecco's Modification of Eagle's Media (DMEM) with 4.5 g/L glucose, 2 mM L-glutamine, and 1 mM sodium pyruvate (Mediatech, Inc., Herndon, VA) and supplemented with 5% fetal bovine sera, 5% Serum supreme, and antibiotics (100 u/L penicillin, 100 ug/L streptomycin, 0.25 ug/L amphotericin B) (Cambrex, East Rutherford, NJ). For infection, cells were incubated in the absence of sera exactly as described and exposed to virus for 1 h (multiplicity of infection (MOI) = 2) [23, 35, 36].

### 2.2. Synthesis and Purification of NSP4 and Cav-1 Peptides

All peptides (Table 1) were synthesized by fluorenylmethoxycarbonyl (Fmoc) solid-phase chemistry with either 1-hydroxy-benzotriazole (HOBt), O-Benzotriazole-N,N,N′,N′-tetramethyluronium-hexafluoro-phosphate (HBTU) and N,N-diisopropylethylamine (DIPEA), or HOBt and N,N′-diisopropylcarbodiimide (DIPCIDI) activation using the Model 90 Peptide Synthesizer (Advanced Chemtech; Louisville, KY). Following synthesis, the peptides were cleaved from the solid resin support and all side-chain protecting groups were removed by the addition of Reagent R (90% trifluoroacetic acid (TFA), 5% thioanisole, 3% ethanedithiol, and 2% anisole). The peptide/Reagent R cleavage mixture was incubated at RT for a maximum of 2 hours with gentle mixing.

To separate the peptides from the solid support, the mixture was filtered through a sintered glass filter into a 50 mL conical tube containing cold diethyl ether in a dry ice/ethanol bath. Following 2-3 rinses of the filter with TFA, the ether solution containing the precipitated peptide was centrifuged at 300 g for 4 minutes. The supernatant was removed and additional cold diethyl ether was added to the peptide pellet. The mixture was allowed to cool in the dry ice/ethanol bath for several minutes before repelleting. This step was repeated an additional two times; the crude peptide was dried under N_2_, resolubilized in 10% acetic acid and lyophilized.

The lyophilized crude peptide was resuspended in 5–10% acetic acid and purified from organic contaminants and incomplete peptide fragments by gravimetric gel filtration chromatography (Sephadex G25 medium). The eluted peptide was lyophilized and further purified by reverse-phase HPLC using either a reverse phase C4 Delta Pak column (Waters Chromatography Division, Milford, MA) or a reverse phase C18 column (Beckman-Coulter, Fullerton, CA) depending on the hydrophobicity of the peptide. Eluted peaks were lyophilized and characterized by matrix-assisted laser desorption/ionization (MALDI) mass spectrometry (Laboratory for Biological Mass Spectrometry, Department of Chemistry, Texas A&M University, College Station, TX). 

### 2.3. Preparation of Small Unilamellar Vesicles (SUV)

Small unilamellar vesicles (SUVs) composed of the neutral lipid POPC, cholesterol, and the anionic phospholipid DOPS in the molar ratio 55 : 35 : 30 were prepared following a previously published protocol [33]. This membrane composition was chosen as a result of previous data showing that this composition and curvature promote an interaction with NSP4-specific peptides as observed by an increase in *α*-helix formation [32, 33]. Stock solutions of the lipids dissolved in chloroform were mixed together in the proper ratio in an amber glass vial. The solvents were removed under N_2_ with constant rotation so that the dried lipids formed a thin film on the wall of the glass vial. The vial containing the dried lipids was further dried under vacuum for a minimum of 4 h. The dried lipids were resuspended in 10 mM MOPS buffer, pH 7.4 (filtered through a 0.45 *μ*m filter; Millipore, Bedford, MA), vortexed and bath sonicated. The resulting multilamellar membrane suspension was sonicated with a microprobe under N_2_ at 4°C at 2 min intervals, followed by 1 min pauses to prevent overheating of the lipid solution. The sonicated lipid solution then was centrifuged at 110,000 g for 4 h to remove any multilamellar vesicles and titanium debris from the sonicator probe. The lipid concentration of the SUV solution was determined by a standard phosphate assay as described [2]. Briefly, the SUV solutions were mixed with a 10% magnesium nitrate (Mg(NO_3_)_2_) solution and heated for 30 min at 120°C. Each sample was ashed in a flame, 600 *μ*L of 0.5 N HCl was added, and the samples were heated in boiling water for 15 min. Each sample was mixed with 1400 *μ*L of a 10% ascorbic acid solution that contained 6 parts of a 0.42% ammonium molybdate solution in 1 N sulfuric acid (H_2_SO_4_) and incubated for 20 min at 45°C before reading the absorbance at 660 nm (A_660_). Lipid concentrations of the SUVs were determined by comparison to a standard curve of 0–200 nmol phosphate.

### 2.4. Direct Fluorescence-Binding Assays

#### 2.4.1. Direct Peptide-Peptide Interaction

A fluorescence-binding assay was utilized to investigate direct binding of the NSP4_112-140_ peptide with each of the cav-1 peptides (N-Cav_2-20_, N-Cav_19-40_, Cav_68-80_, and C-Cav) [20, 39]. All of the cav-1 peptides directly were labeled with a Cy3 fluorescent dye, using the Cy3 Monoreactive dye pack from Amersham Biosciences according to the manufacturer's protocol. Briefly, each peptide was dissolved in 50 nM phosphate buffer, pH 7.0, at a concentration of 1 mg/mL, and added to a vial containing lyophilized Cy3 dye. The solution gently was mixed and incubated at 25°C for at least 2 h with intermittent mixing. Labeled peptides were separated from unlabeled peptides by Sephadex G25 gel filtration chromatography.

To demonstrate a direct peptide-peptide interaction between the NSP4_112-140_ peptide and each of the cav-1 peptides, increasing concentrations of NSP4_112-140_ (10–500 nM) were added to 25–100 nM of the Cy3-labeled cav-1 peptides in 2 mL of phosphate-buffered saline (PBS, pH 7.4) in either the absence or presence of small unilamellar vesicles (SUVs, 1 *μ*M). The Cy3 fluorophore was excited at 550 nm and emission spectra were scanned from 560 to 700 nm. Fluorescence emission spectra were obtained at 25°C with a PC1 photon-counting spectrofluorometer with excitation and emission slit widths of 1.0 (ISS, Inc.). Spectra were corrected for background and maximal fluorescence intensities were recorded. Calculation of the dissociation constants (*K*_*d*_) was performed from the titration curves plotted as quenching in Cy3-peptide fluorescence intensity (*F*_0_ − *F*, where *F*_0_ and *F* represented the Cy3-cav-1 peptide fluorescence intensities in the absence and presence of NSP4_112-140_ or cav-1 peptide, resp., at each titration point) as a function of peptide concentration. The *K*_*d*_ also was obtained by a reciprocal plot of 1/(1 − *F*/*F*_max⁡⁡_) and *C*_*L*_/*F*/*F*_max⁡_ according to the following equation: *y* = *bx* + *y*_0_, where *F* is the fluorescence intensity at a given concentration of ligand, *F*_max⁡⁡_ is the maximal fluorescence obtained, and *C*_*L*_ is the ligand concentration. The slope of the line (b) is equal to 1/*K*_*d*_.

#### 2.4.2. Cholesterol-Peptide Interaction

Similarly, the NSP4_112-140_-cholesterol interaction and binding affinity were determined by a direct fluorescent binding assay in which the peptide was labeled with a Cy5 fluorophore using the Cy5 Monoreactive dye pack from Amersham Biosciences (Piscataway, NJ). A concentrated cholesterol stock solution (20 uM) was prepared by dissolving the cholesterol in ethanol. To analyze a binding interaction between NSP4_112-140_ and cholesterol, an increasing quantity of cholesterol (5–35 nM) was added to 10 nM of the Cy5-labeled NSP4_112-140_ in 2 mL of PBS (pH 7.4). The Cy5 fluorophore was excited at a wavelength of 649 nm, and the emission spectra were scanned from 655 to 720 nm using a PCI photon-counting spectrofluorometer (ISS, Inc., Champaign, IL). Fluorescence emission spectra were obtained at 25°C with excitation and emission slit widths of 1.0. As with the peptide-peptide interactions, spectra were corrected for background and maximal fluorescence intensities were measured. Calculation of the *K*_*d*_ was determined from the plotted titration curves whereby the Cy5-peptide fluorescence intensity was quenched and measured as a function of cholesterol concentration (*F*_0_−*F*, where *F*_0_ and *F* represented the Cy5- NSP4_112-140_ fluorescence intensities in the absence and presence of cholesterol, resp., at each titration point). The sigmoidal curves also were fitted to a Hill plot according to the following equation: *y* = *ax* ^*b*^/(*c* ^*b*^ + *x* ^*b*^), where *y* and *x* correspond to (*F*_0_−*F*) and the ligand concentration at each point, while *a*, *b*, and *c* represent the maximum binding (*B*_max⁡⁡_), the number of binding sites (*n*), and the *K*_*d*_ value, respectively. *K*_*d*_ values were calculated with the sigmoidal function of Sigma Plot (SPSS, Chicago, IL) utilizing the Hill plot feature.

### 2.5. Circular Dichroism (CD) and Secondary Structure Estimations

The secondary structures of the NSP4 and cav-1 specific peptides were determined by circular dichroism following a previously published protocol [32, 33]. Briefly, each peptide (15–35 *μ*M) was suspended in 10 mM potassium phosphate buffer, pH 7.4, or 50% trifluoroethanol (TFE) to promote a hydrophobic environment and folding of the peptide, in the presence or absence of lipid vesicles (1 mM). Samples containing buffer or SUVs without peptide were used for background correction. Peptide concentrations were determined by amino acid analysis (Protein Chemistry Laboratory, Department of Biochemistry, Texas A&M University, College Station, TX). CD spectra were obtained in a 1 mm circular quartz cell using a Model J-710 JASCO spectropolarimeter (JASCO, Easton, MD) or in a 1 mm rectangular quartz cell in a Model 202 Aviv spectrometer (Aviv Biomedical, Lakewood, NJ). The spectra for each of the peptides were recorded from 185 nm to 260 nm, with a step resolution of 1 nm, speed of 50 nm/min, response of 1 sec, bandwidth of 2.0 nm, and sensitivity of 10 mdeg. Data were averaged from 5 scans, background subtracted, smoothed, and converted into mean residue molar ellipticity [*θ*] (deg cm^2^/dmol).

To determine the percent *α*-helix for each of the NSP4- and cav-1 specific peptides, the following equation was used: *θ*_222_ = (*f*_*h*_ − *ίκ*/*N*)[*θ*_*h*  222*∞*_] [12, 21, 22]. In this equation, *θ*_222_ is the mean residue molar ellipticity at 222 nm, *f*_*h*_ is the fraction in *α*-helical form, *ί* is the number of helices, *κ* is a wavelength-specific constant with a value of 2.6 at 222 nm, *N* is the number of residues in the peptide, and *θ*_*h*  222*∞*_ is the molar ellipticity for a helix of infinite length at 222 nm, that is, −39,500 deg cm^2^/dmol.

### 2.6. Treatment of RV-Infected MDCK and -HT29.f8 Cells with Cholesterol-Altering Drugs

MDCK cells were infected with RV at a multiplicity of infection (MOI) of 2 and incubated for 12 hpi. The cells were washed and treated in triplicate with the cholesterol altering drugs, fillipin [40], and nystatin [41] exactly as described. Chlorpromazine [40, 42] and nocodazole [39] were utilized as negative and positive controls, respectively. Cells were fixed with methanol : acetone (1 : 1) at −20°C for 10 min, blocked with 5% dry milk for 20 min, incubated with anti-NSP4_150-175_ and anti-rabbit-IgG-FITC, and examined under a UV microscope (Zeiss). The cell surface carefully was observed and the presence or absence of fluorescence carefully noted. Comparisons also were made to untreated cells. Primary and secondary antibody controls were negative. To ensure the lack of drug toxicity, a separate set of cells were treated and then stained with trypan blue.

### 2.7. Direct Fluorescence-Binding Assays

#### 2.7.1. NSP4_112-140_ Binds the N-Terminus of cav-1 (Cav_19-40_) with Greater Affinity Than the C-Terminus

We previously demonstrated that NSP4_114-135_ interacts with both the N- and C-termini of cav-1 using yeast-2-hybrid and pull-down assays, which is unusual [24]. To further investigate this finding and determine if there was a preferential binding of NSP4 to the N- or C-terminus of cav-1, the NSP4_112-140_ peptide that encompasses aa 114–135 and various cav-1 peptides (N-Cav_2-20_, N-Cav_19-40_, and C-Cav_161-178_) were utilized in a direct fluorescence-binding assay.

**Figure 1:**
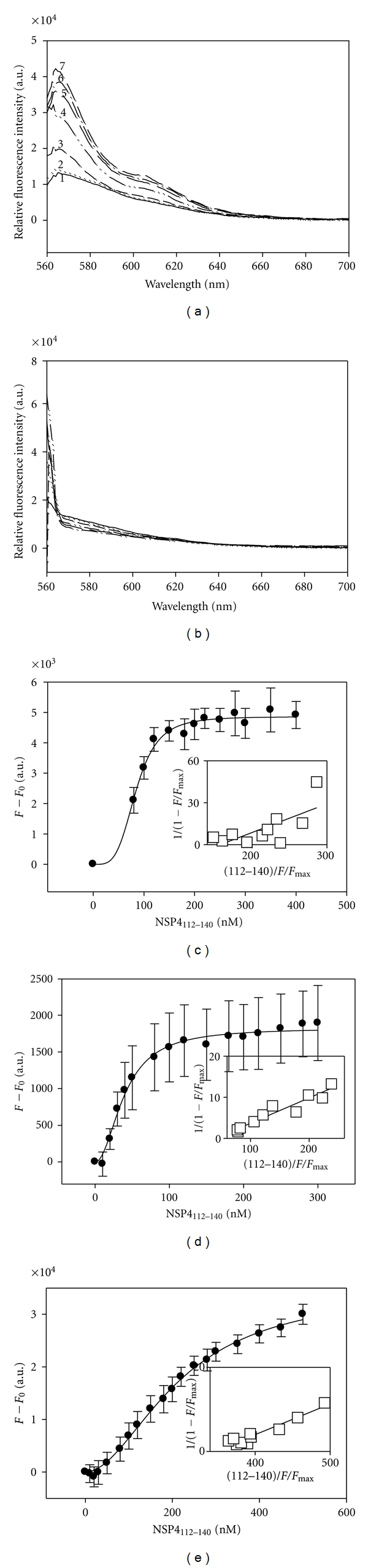
Direct Fluorescence-Binding Assays of NSP4_112-140_ with Caveolin-1 Peptides. (a) Fluorescence spectra of Cy3-C-Cav_161-178_ titrated with increasing concentrations of NSP4_112-140_. *Spectrum *1 : 100 nM Cy3-C-Cav_161-178_ in buffer only. *Spectra *2–11 : 100 nM Cy3-C-Cav_161-178_ in the presence of 10, 100, 150, 200, 250, 300, 350, 400, 450, and 500 nM of NSP4_112-140_, respectively. (b) Fluorescence spectra of Cy3-C-Cav_161-178_ titrated with increasing concentrations of NSP4_150-175_ (negative control). Plots (c)–(e) show maximal fluorescence emission (measured at 565 nm upon excitation at 550 nm) for NSP4_112-140_ in the presence of 25 nM Cy3-N-Cav_2-20_ (c), 50 nM Cy3-N- Cav_19-40_ (d), and 100 nM Cy3-C-Cav_161-178_. (e). *Insets*: linear plots of the binding curve of NSP4_112-140_.

 Upon titration with increasing concentrations of NSP4_112-140_, each of the Cy-3-labled N- or C-terminal cav-1 peptides showed an increase in fluorescence intensity at 565 nm, indicative of an interaction. Plots of NSP4_112-140_ concentrations versus maximum fluorescence intensities revealed saturated binding curves for each of the cav-1 peptides (Figure 1). Linear reciprocal plots were used to calculate the *K*_*d*_ for each peptide-peptide pair (Table 2). The calculated *K*_*d*_ values indicated that NSP4_112-140_ bound the N-Cav_19-40_ peptide with the strongest affinity (40 ± 10 nM), while it bound the C-Cav_161-178_ peptide with the weakest affinity (217 ± 35 nM). The N-Cav_2-20_ peptide bound with intermediate affinity (85 ± 6 nM). None of the cav-1 peptides bound to the control peptide corresponding to the C-terminus of NSP4 (NSP4_150-175ΔAla_), which is known to not bind cav-1 [23]. It is unusual for a protein to interact with both termini of cav-1. We now show that there is a preferential interaction of NSP4_112-140_ with the N-terminus of cav-1, which, to our knowledge, is the first report of a preferential binding to one termini of cav-1.

**Figure 2:**
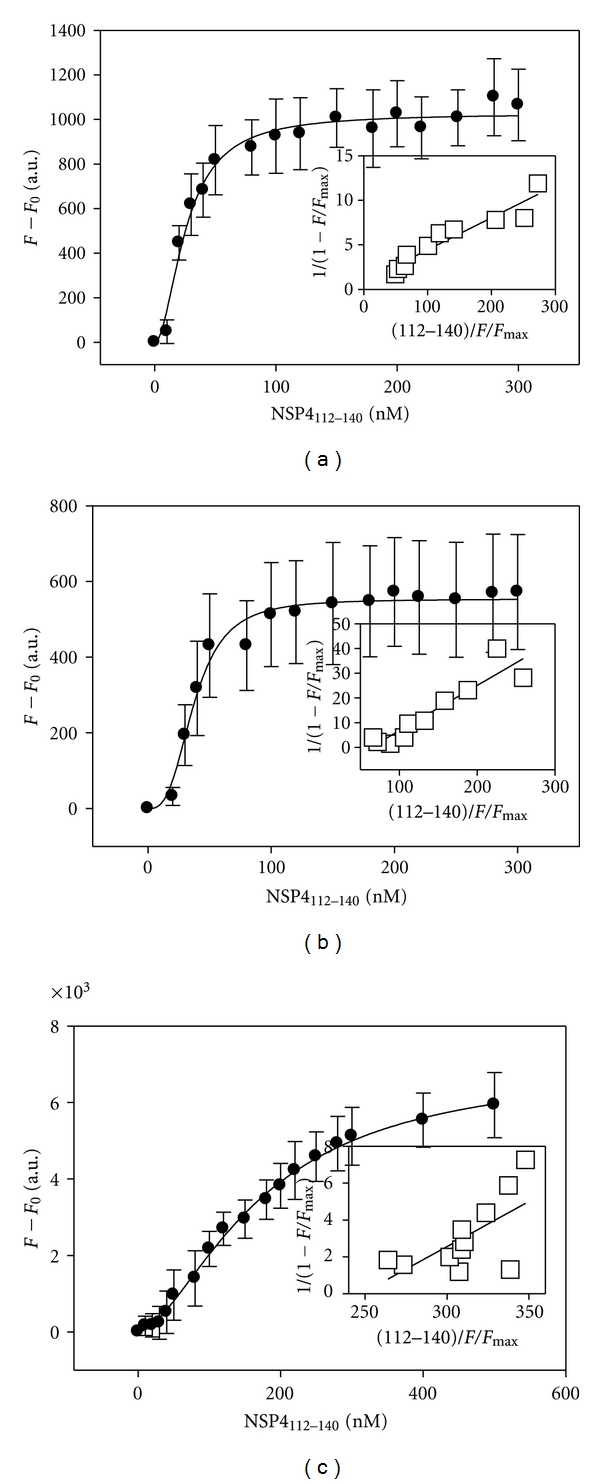
Direct Fluorescence-Binding Assays of NSP4_112-140_ with Caveolin-1 Peptides in the presence of 1 *μ*M SUV model membranes. Direct binding based on dequenching of Cy3 fluorescence emission. (a–c) Plots of maximal fluorescence emission (measured at 565 nm upon excitation at 550 nm) for NSP4_112-140_ in the presence of 1 uM SUV and either 25 nM Cy3-N-Cav_2-20_ (a), 50 nM Cy3-Cav_19-40_ (b), or 100 nM Cy3-C-Cav_161-178_ (c). *Insets*: linear plots of the binding curve of NSP4_112-140_.

Previous CD analyses of NSP4 peptides corresponding to the cav-1 binding domain show that aa 114–135 interacts with model membranes (SUVs) [32, 33]. Therefore, NSP4-cav-1 peptide-peptide interactions also were investigated in the presence of the same SUV membranes. The SUVs were mixed with each of the Cy3-labeled cav-1 peptides in PBS and then titrated with increasing concentrations of NSP4_112-140_ (Figure 2).

 The presence of lipid vesicles had no effect on the interaction of NSP4_112-140_ with either N-Cav_19-40_ or C-Cav_161-178_ (Figures 2(b) and 2(c)). Binding affinities in the presence of membranes were similar to those calculated in the absence of membranes (Table 2). However, the presence of the SUVs resulted in an increased affinity between NSP4_112-140_ and N-Cav_2-20_. The calculated *K*_*d*_ value was determined to be 26 ± 4 nM, an approximate 3-fold increase over that observed in the absence of membranes (85 ± 6 nM) and about a1.5-fold increase over the *K*_*d*_ of N-Cav_19-40_ in the absence of membranes (40 ± 10 nM). These results strongly suggest that the presence of lipid vesicles enhances binding between NSP4_112-140_ and the extreme N-terminus of cav-1 (aa 2-20), but not the adjacent N-terminal cav-1 peptide, N-Cav_19-40_.

#### 2.7.2. The N-terminal cav-1 Peptides (aa 2–20 and 19–40) Failed to Bind the cav-1 C-Terminal Peptide (aa 161–178)

**Figure 3:**
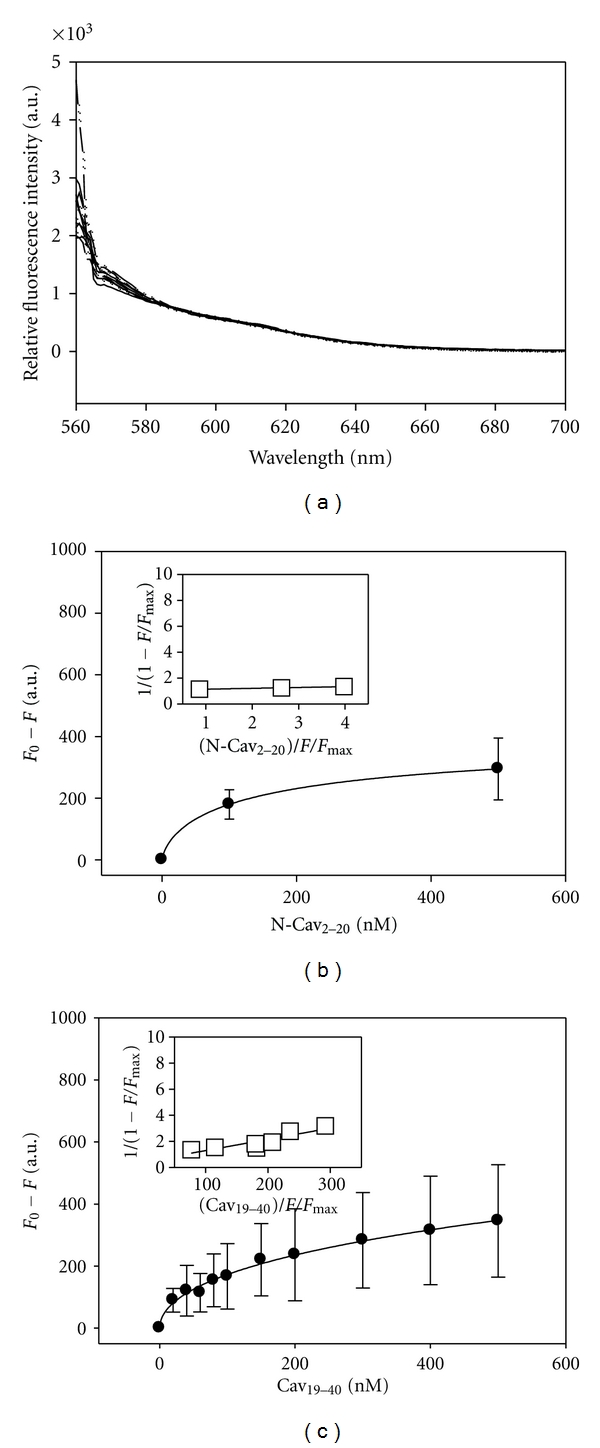
Direct Fluorescence-Binding Assays of N-Cav_2-20_ or N-Cav_19-40_ Peptide with C-Cav_161-178_ Peptide. (a) Fluorescence spectra of Cy3-C-Cav_161-178_ titrated with increasing concentrations of Cav_19-40_ (0–500 nM). (b-c) Plots of maximal fluorescence emission (measured at 565 nm upon excitation at 550 nm) for (b) N-Cav_2-20_ and (c) N-Cav_19-40_ in the presence of 100 nM Cy3-C-Cav_161-178_. *Insets*: linear plots of the binding curve of (b) N-Cav_2-20_ and (c) N-Cav_19-40._

To evaluate the mechanism of the interaction of NSP4 with both cav-1 termini, we examined whether the two cav-1 termini bound one another to facilitate the interaction with NSP4 by employing the fluorescence-binding assay. C-Cav_161-178_ was labeled with a Cy3-fluorophore and titrated with either the N-Cav_2-20_ or N-Cav_19-40_ peptide. Titration of C-Cav_161-178_ with increasing concentrations of either N-terminal peptide resulted in no change in fluorescence intensity at 565 nm, indicative of a lack of association (Figure 3). Hence, we propose that the N- and C- termini of cav-1 do not bind one another and the interaction between NSP4 and the cav-1 termini does not result from an initial binding between the cav-1 N- and C-termini. To our knowledge, this is the first report of the cav-1 termini not interacting. Additional studies are needed to dissect the mechanism and implications of both cav-1 termini binding NSP4.

### 2.8. Circular Dichroism Analysis

#### 2.8.1. Secondary Structure of NSP4_112-140_

**Figure 4:**
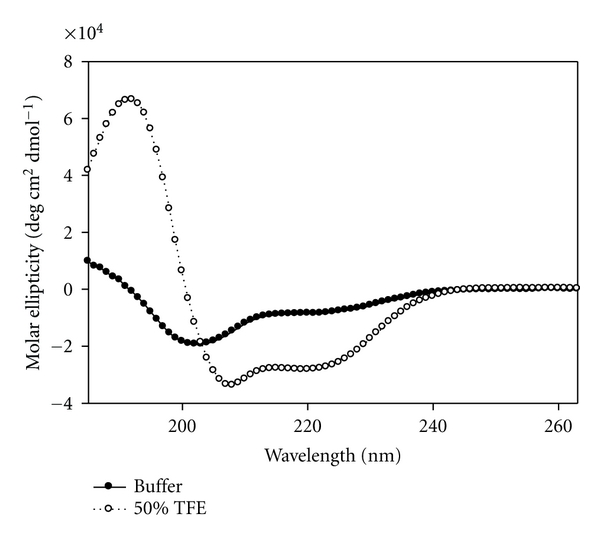
CD spectra of NSP4_112-140_ in aqueous buffer (●) and 50% TFE (∘).

The structure of the NSP4_112-140_ peptide was first determined in an aqueous buffer (10 mM potassium phosphate buffer, pH = 7.4) (Figure 4, dark circles). The CD spectrum showed double minima at 208 and 222 nm and a single maximum peak at 190 nm, indicative of some *α*-helical secondary structure. Using the molar ellipticity value at 222 nm [43], the *α*-helical content of the NSP4_112-140_ peptide was calculated to be 30.6 ± 2.4%.

 To enhance innate peptide structure, the secondary structure of NSP4_112-140_ was determined in 50% TFE, a hydrophobic solvent that promotes intramolecular hydrogen bonding (Figure 4, open circles). The CD spectrum showed a dramatic increase in *α*-helix formation of the peptide, as evidenced by an increase in both the negative molar ellipticity at 208 and 222 nm and the positive molar ellipticity at 190 nm. The *α*-helical content was calculated using the molar ellipticity value at 222 nm and determined to be 79.6 ± 4.6% (Table 3).

 Previous CD experiments with the enterotoxic peptide, NSP4_114-135_ demonstrate that the *α*-helical content increases in the presence of SUVs containing POPC, cholesterol, and DOPS at a 55 : 35 : 10 molar ratio [32, 33]. To determine the secondary structural changes that occur in the presence of membranes, NSP4_112-140_ was mixed with SUV membranes and changes in *α*-helical content were noted by CD.

**Figure 5:**
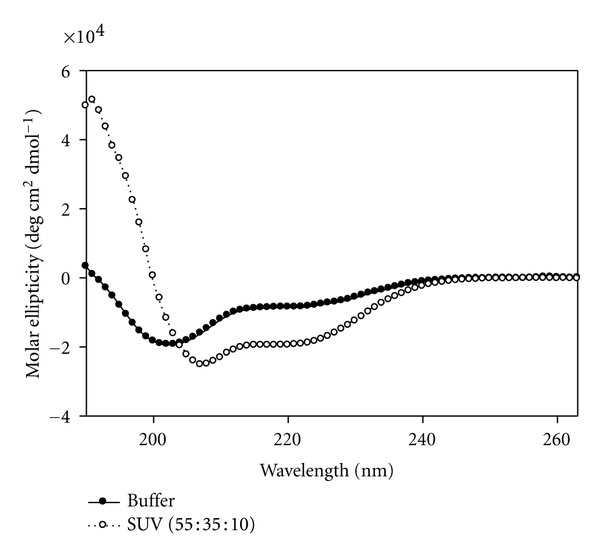
CD spectra of NSP4_112-140_ in aqueous buffer (●) and in the presence of 1 mM SUV model membranes (55 : 35 : 10, POPC/cholesterol/DOPS) (∘).

The CD spectrum of the NSP4_112-140_ peptide in the presence of 1 mM SUV showed nearly a 2-fold increase in *α*-helix formation over that in aqueous buffer, as evidenced by the increase in both the negative molar ellipticity as 208 and 222 nm and the positive molar ellipticity at 190 nm (Figure 5). The *α*-helical content was calculated using the molar ellipticity value at 222 nm and determined to be 57.9 ± 1.6% (Table 3).

#### 2.8.2. Secondary Structure of Caveolin-1 Peptides

**Figure 6:**
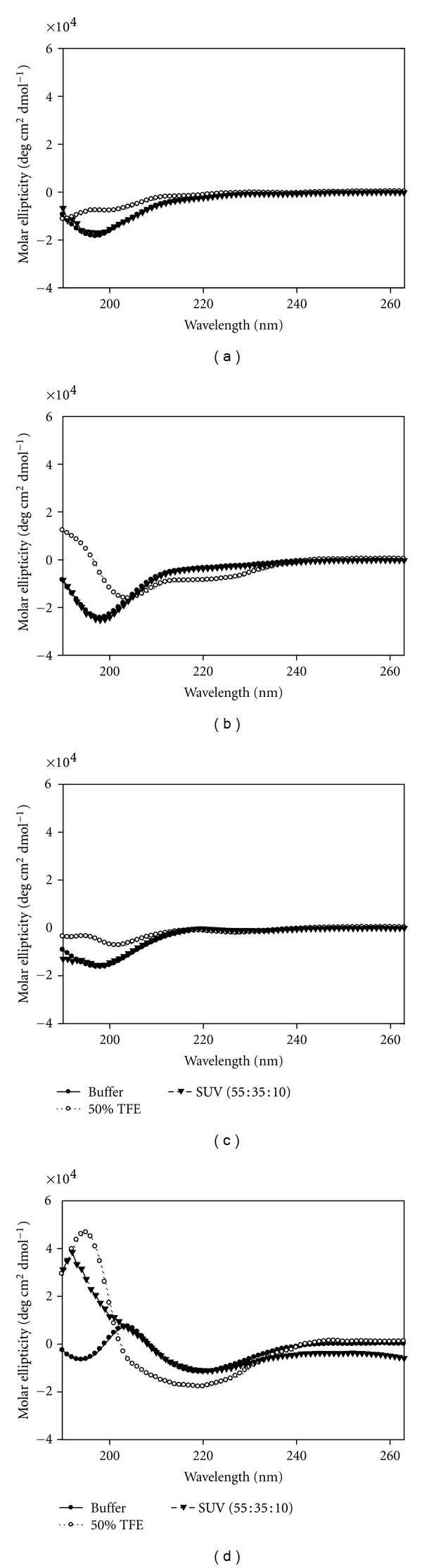
CD Spectra of cav-1 peptides in aqueous buffer (●), 50% TFE (∘), and in the presence of 1 mM SUV (55 : 35 : 10, POPC/cholesterol/DOPS) (*▼*). (a) N-Cav_2-20_; (b) N-Cav_19-40_; (c) Cav_68-80_; (d) C-Cav_161-178_.

The secondary structures of the cav-1 peptides were determined and evaluated in the presence of 50% TFE and 1 mM SUVs (55 : 35 : 10; POPC : Cholesterol : DOPS) by CD (Table 3). In aqueous buffer, the *α*-helical content of N-Cav_2-20_, N-Cav_19-40_, and Cav_68-80_ was quite similar, at 18.9%, 20.7%, and 23.4%, respectively (Figures 6(a)–6(c), dark circles). The *α*-helical contents of the C-terminal peptide (C-Cav_161-178_) was approximately twice (43.4%) that of the N-terminal peptides (Table 3). When placed in 50% TFE, the *α*-helical content of N-Cav_2-20_, N-Cav_19-40_, Cav_68-80_, and C-Cav_161-178_ peptides were calculated to be 16.5%, 33.4%, 23.1%, and 67.1% respectively (Figure 6, open circles). Only N-Cav_19-40_ and C-Cav_161-178_ showed an increase in *α*-helix formation in the presence of 50% TFE.

When mixed with the SUVs, none of the cav-1 peptides demonstrated a change in *α*-helix formation over that seen in aqueous buffer (Figure 6, dark triangles). This result was anticipated as none of these peptides corresponded to a cav-1 membrane interacting or transmembrane domain.

#### 2.8.3. Secondary Structural Changes Observed upon Mixing NSP4_112-140_ with C-Cav_161-178_

Analyses by direct fluorescence-binding assays revealed that N-Cav_19-40_ bound NSP4_112-140_ with a stronger affinity than C-Cav_161-178_. We therefore investigated whether structural changes occurred upon the association of the NSP4 and cav-1 peptides, in particular with the amino terminus. Each of the cav-1 peptides (N-Cav_2-20_, N-Cav_19-40_, Cav_68-80_, and C-Cav_161-178_) was mixed with the NSP4_112-140_ peptide and was analyzed by CD. Cav_68-80_ was used as a negative control, as previous studies have shown that NSP4 does not interact with this region of cav-1 [23, 24]. Analyses of the mixing experiments were achieved by comparing the spectrum of each peptide-peptide mixture (observed) with the sum of the individual spectra of each peptide (theoretical). The *α*-helical content was calculated and significant differences (Student's *t*-test, *P* < 0.05) between the observed and theoretical values indicated a change in secondary structure and binding between the two peptides. However, a lack of secondary structural alterations upon mixing the peptides did not negate binding between the NSP4_112-140_ and the cav-1 peptide being tested. While direct fluorescent binding assays revealed the affinity of each of the peptide-peptide interactions, the CD experiments disclosed the extent to which the interaction(s) caused a change in the secondary structure.

**Figure 7:**
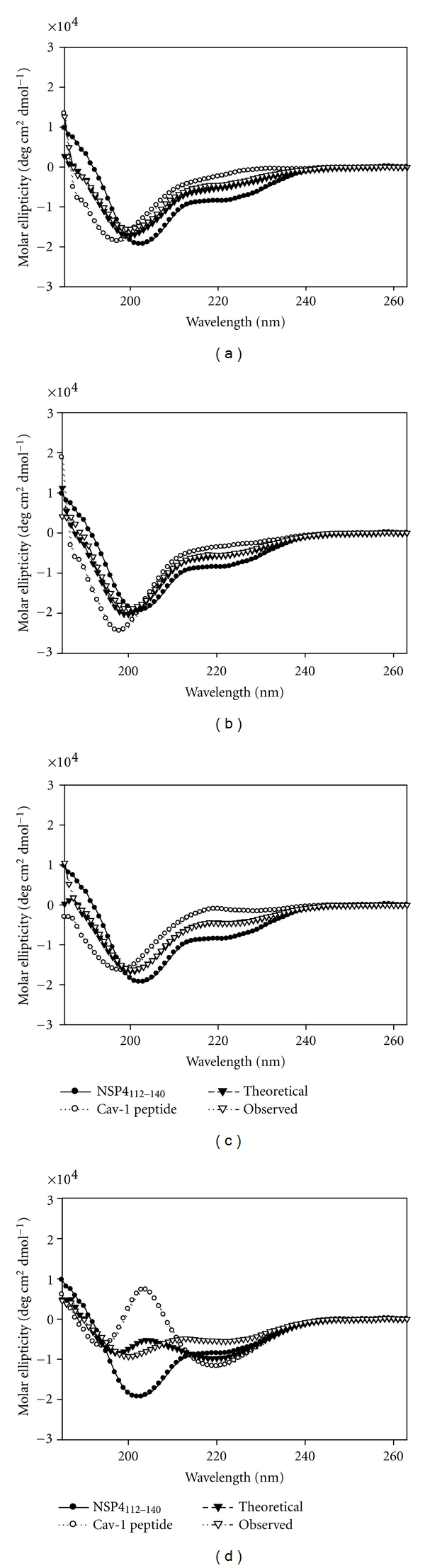
CD spectra of NSP4_112-140_-caveolin-1 peptide-peptide interactions in aqueous buffer. (a) NSP4_112-140_ + N-Cav_2-20_; (b) NSP4_112-140_ + Cav_19-40_; (c) NSP4_112-140_ + Cav_68-80_; (d) NSP4_112-140_ + C-Cav_161-178_. (●) NSP4_112-140_ spectra; (∘)-Cav-1 peptide spectra; (*▼*) Theoretical spectra; (Δ) Observed spectra peptide-peptide combination was mixed with the SUVs, and any alteration in structure was noted.

 When each of the cav-1 peptides was mixed with NSP4_112-140_ in an aqueous buffer, only the NSP4_112-140_-C-Cav_161-178_ mixture showed a significant difference between the observed and theoretical values (25.0 ± 1.7% versus 35.3 ± 1.6%  *α*-helix) (Figure 7(d); Table 4). No change in secondary structure was observed for the NSP4_112-140_-N-Cav_2-20_ (21.7 ± 0.8% versus 23.6 ± 2.6%), the NSP4_112-140_-N-Cav_19-40_ (23.8 ± 1.2% versus 24.4 ± 0.6%), or the NSP4_112-140_-Cav_68-80_ negative control (24.2 ± 0.3% versus 23.9 ± 0.7%) mixtures even though N-Cav_19-40_ had the lowest *K*_*d*_ (Figures 7(a)–7(c); Table 4). Similarly, when each peptide-peptide pair was placed in 50% TFE, only the NSP4_112-140_-C-Cav_161-178_ mixture showed a significant change in secondary structure (44.2 ± 10.6% versus 67.8 ± 3.2%) (Table 4). These data confirmed the weak binding between NSP4_112-140_ and C-Cav_161-178_ and verified that secondary structure alterations do not always occur upon an interaction.

### 2.9. Effect of SUV Model Membranes on the NSP4_112-140_-Caveolin-1 Peptide-Peptide Interactions

**Figure 8:**
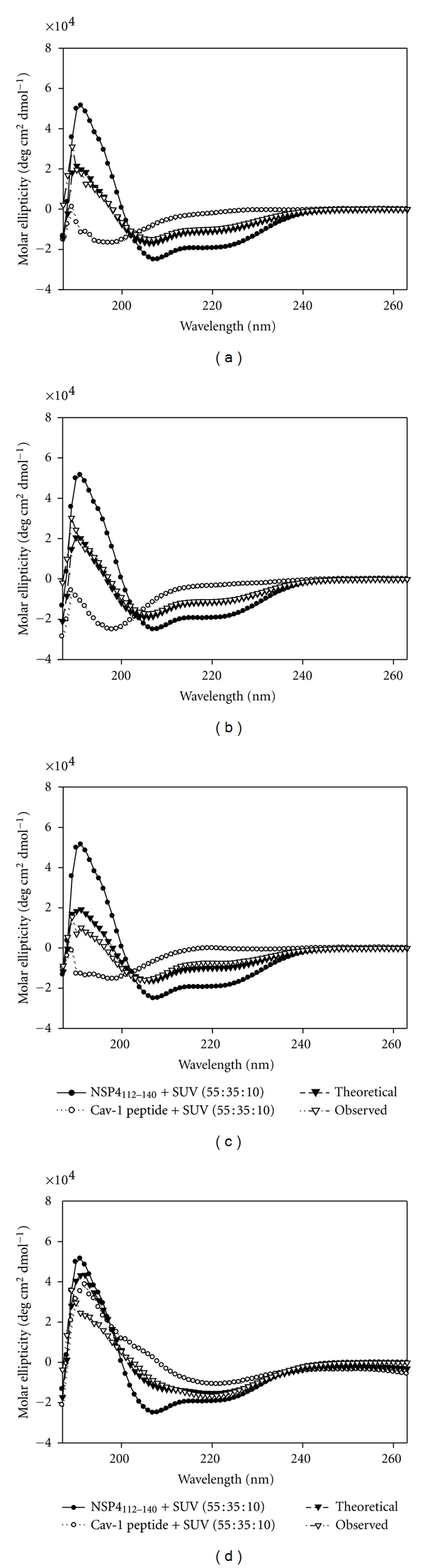
CD spectra of NSP4_112-140_-Cav-1 peptide-peptide interactions in the presence of 1 mM SUV (55 : 35 : 10, POPC/cholesterol/DOPS) in aqueous buffer. (a) NSP4_112-140_ + N-Cav_2-20_; (b) NSP4_112-140_ + Cav_19-40_; (c) NSP4_112-140_ + Cav_68-80_; (d) NSP4_112-140_ + C-Cav_161-178_. (●) NSP4_112-140_ spectra; (∘) Cav-1 peptide spectra; (*▼*) Theoretical spectra; (Δ)-Observed spectra.

Cav-1 is critical to both the structure and function of caveolae, is the major constituent of caveolae, and is controller of caveolae formation [44]. NSP4 is present in isolated caveolae from RV-infected cells [34], and together with NSP4_114-135_, preferentially interacts with caveolae-like model membranes [32]. Therefore we evaluated the extent the SUVs influenced secondary structure alterations of the NSP4-cav-1 peptide-peptide interactions. Each NSP4_112-140_-cav-1 peptide pair was monitored by CD (Figure 8).

Even though the NSP4_112-140_-C-Cav_161-178_ peptide pair showed the most change in *α*-helical content in aqueous buffer and 50% TFE, in the presence of SUVs there was not a significant conformational change indicating that this interaction was not enhanced by the presence of model membranes (Table 4). Similarly, the NSP4_112-140_-N-Cav_19-40_ showed the strongest affinity by direct fluorescent binding assays (Table 2), yet this interaction failed to result in a significant change in secondary structure when mixed with membranes. The observed *α*-helical content did not vary from the expected theoretical value, regardless of whether the peptide mixture was in aqueous buffer, 50% TFE, or mixed with SUVs. These results suggest that the strong binding between NSP4_112-140_ and N-Cav_19-40_ required neither a change in structure nor the presence of specific lipids.

Only the NSP4_112-140_-Cav_68-80_ (negative control) mixture showed a significant change in the *α*-helical content in the presence of the SUVs (31.3 ± 0.2% versus 37.5 ± 1.9%) (Figure 8(c); Table 4). It is currently unclear why the control peptide mixture resulted in an alteration in helical structure and requires further study.

### 2.10. NSP4_112-140_ Peptide-Cholesterol Interaction

The NSP4_112-140_ peptide, which contains the CRAC motif, was labeled with a Cy5-fluorophore and titrated with cholesterol in a direct fluorescence-binding assay. The maximum solubility of cholesterol in water is 4.7 *μ*M and the critical micellar concentration (CMC) is 25–40 nM [18], so cholesterol concentrations were kept below these limits in the binding assays.

**Figure 9:**
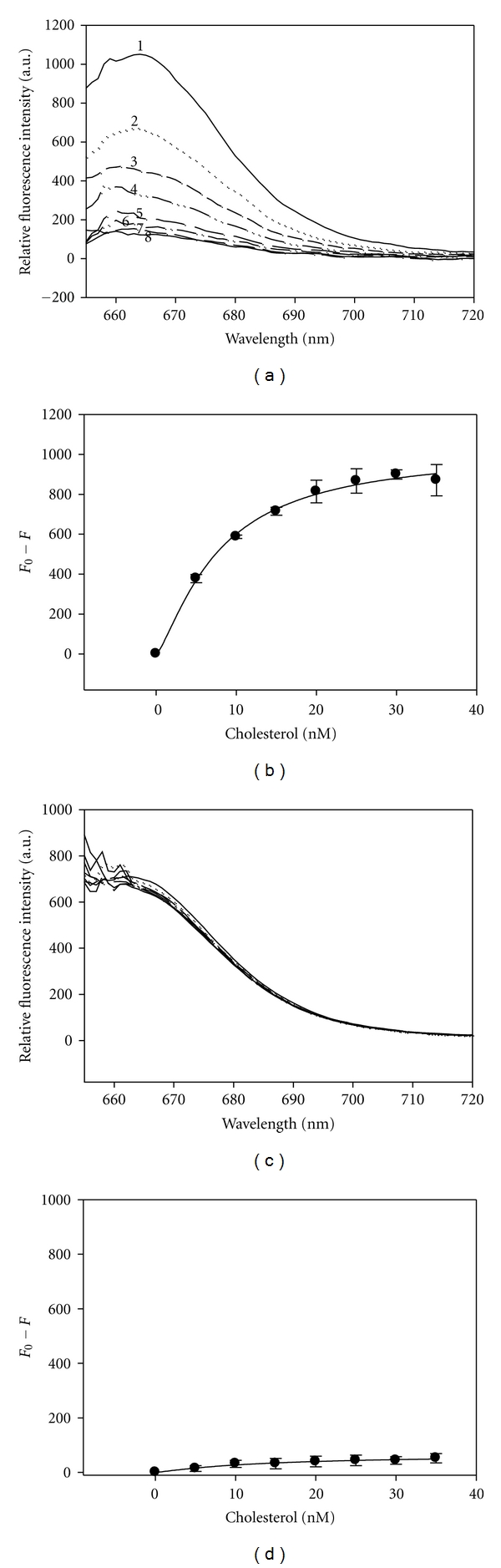
Cholesterol directly binds NSP4_112-140_ peptide. (a) Fluorescence spectra of Cy5-NSP4_112-140_ titrated with increasing concentrations of cholesterol. *Spectrum *1 : 10 nM Cy5-NSP4_112-140_ in buffer only. *Spectra *2–8 : 10 nM Cy5-NSP4_112-140_ in the presence of 5, 10, 15, 20, 25, 20 and 35 nM of cholesterol, respectively. (b) Plot of maximal fluorescence emission (measured at 665 nm upon excitation at 649 nm) for cholesterol in the presence of 10 nM Cy5-NSP4_112-140_. Values represent the mean ± S.E., *n* = 4. (c) Fluorescence spectra of Cy5-NSP4_150-175ΔAla_ (negative control) titrated with increasing concentrations of cholesterol. (d) Plot of maximal fluorescence emission (measured at 665 nm upon excitation at 649 nm) for cholesterol in the presence of 10 nM Cy5-NSP4_150-175ΔAla_. Values represent the mean ± S.E., *n* = 4.

 In the absence of cholesterol, the Cy5-NSP4_112-140_ peptide showed maximum fluorescence at 665 nm (Figure 9). Upon titration with increasing concentrations of cholesterol (5–35 nM), Cy5-NSP4_112-140_ showed a decrease in fluorescence intensity, indicative of an interaction. Measurements of fluorescent intensities at 665 nm of Cy5-NSP4_112-140_ in the presence of increasing concentrations of cholesterol were corrected for background (Cy5-NSP4_112-140_ in buffer alone) and plotted as a function of cholesterol concentration, demonstrating a saturable binding curve (Figure 9(b)). The *K*_*d*_ of cholesterol for NSP4_112-140_ was calculated to be 8 ± 1 nM. A Hill plot revealed a single cholesterol binding site (*n* = 1) in the 28 residue peptide. A control peptide (NSP4_150-175ΔAla_), corresponding to the C-terminus of NSP4 and lacking the CRAC motif, failed to demonstrate a change in fluorescence intensity in the presence of cholesterol, indicative of a lack of binding (Figures 9(c) and 9(d)).

### 2.11. Cholesterol Altering Drugs Disrupt NSP4 Transport

Specific inhibitors aid in the dissection of biological processes. We evaluated the effect(s) of drugs known to disrupt cholesterol (fillipin, nystatin, lovastatin) [40, 41, 45], hence caveolin/caveolae transport properties [46], an inhibitor of clathrin-coated pit processes (chloropromazine) [40, 42], and a disruptor of the cytoskeleton (nocodazole) [39]. Cells were treated at the recommended effective dose, evaluated with trypan blue to ensure a lack of toxicity, and NSP4 transport to the cell periphery was noted. Whenever cholesterol trafficking was disrupted, NSP4 likewise failed to transport to the cell surface as evidenced by a lack of peripheral staining (Table 5).

## 3. Discussion

Several reports highlight the use of peptides as a means of investigating the interactions of full-length proteins [3, 47–50]. This study similarly demonstrated the utility of synthetic peptides by showing differential binding of NSP4_112-140_ to the cav-1 termini and disclosing secondary structural changes that were not necessary for the interaction between the NSP4 and cav-1 binding domains. We also demonstrated a direct interaction between NSP4 and cholesterol. The *K*_*d*_ was calculated as 8.0 ± 1 nM and a Hill plot revealed a single binding site for cholesterol when reacted with NSP4_112-140_.

An interaction between NSP4_114-135_ and the cellular protein cav-1 [23–25] as well as caveolae is well established [34]. Additionally, we report the interaction of NSP4 with both the N- and C-termini of cav-1 (aa 2–31 and 161–178, resp.) based on yeast two-hybrid analyses and *in vitro* peptide binding assays [24]. Herein, we confirmed the interaction between NSP4 and the N- and C-termini of cav-1 and calculated the binding affinities of those interactions using peptides and direct fluorescence-binding assays. NSP4_112-140_, which encompasses the cav-1 binding domain and overlaps the AAH and enterotoxic peptide, was utilized with cav-1 peptides corresponding to the N- (N-Cav_2-20_ and N-Cav_19-40_) and C- (C-Cav_161-178_) termini and a central region (Cav_68-80_). Calculations of the binding affinities revealed that NSP4_112-140_ bound the N-terminus of cav-1 (N-cav_19-40_) with an affinity over 5 times stronger than that of the C-terminus (C-cav_161-178_) (40 ± 10 nM versus 217 ± 35 nM, resp.). When mixed with SUVs, the *K*_*d*_ values showed an increase in binding affinity for the NSP4_112-140_-N-Cav_2-20_ interaction such that this peptide-peptide interaction occurred with similar affinity (26 ± 4 nM) as that observed between NSP4_112-140_ and N-Cav_19-40_ in the absence of membranes (40 ± 10 nM). No change, however, was observed in the binding affinities between NSP4_112-140_ and N-Cav_19-40_ or C-Cav_161-178_ in the presence of the lipid vesicles indicating that lipids were not involved in these interactions. The functional significance of the preferential binding to N-terminal cav-1 currently is unknown.

While we did not specifically analyze the oligomerization of the NSP4_112-140_ peptide in this study, our previous data on a similar and overlapping peptide (NSP4_114-135_) showed oligomerization/aggregation at a peptide concentration of ~50 uM [32]. The peptide concentration utilized in the binding assays stayed within the nM range, well below the 50 uM concentration at which the oligomerization occurred with the NSP4_114-135_ peptide.

 Following confirmation of an interaction between NSP4 and both termini of cav-1, we analyzed potential secondary structural changes upon peptide-peptide association by CD. Previous secondary structure analysis of NSP4_114-135_ yielded an estimate of 37%  *α*-helix for the peptide [32, 33]. Analyses of the secondary structure of the full-length NSP4 protein showed ~26%  *α*-helix when analyzed by CD [32]. To our knowledge, all other structural studies of NSP4 were completed on specific fragments of NSP4 and not the full-length protein. The C-terminal region of NSP4 clearly is helical [13]. CD analysis of NSP4_112-140_ showed 30%  *α*-helix, which is within the range calculated for the enterotoxic peptide and the full-length protein. When placed in the presence of SUVs, which mimics the more biologically relevant caveolae environment, NSP4_112-140_ demonstrated an increase in *α*-helical content. This increase in *α*-helix formation indicated that this peptide behaved similarly to the NSP4_114-135_ peptide and the presence of membranes influenced the structure.

To date the crystallographic structure of cav-1 has not been resolved; however, limited secondary structural studies and bioinformatics analyses have provided insight into its folded conformation. CD analysis of aa 1–101 reveals that the N-terminus contains 20%  *α*-helix, with aa 79–96 constituting the *α*-helix portion, while aa 1–78 likely lacks significant secondary structure [51]. An additional study, which used a bioinformatics approach, notes that aa 95–101 contains *α*-helical structure, while aa 84–94 forms two *β*-strands [52]. Further, cav-1 is known to form high molecular weight oligomers that requires the cav-1 scaffolding domain (CSD, aa 82–101) [52–54]. Additionally, the C-terminus is palmitoylated on three cysteines, which helps anchor the protein to the membrane and may thus explain the preferential binding of NSP4 to the N-terminus of cav-1 [54–56].

The data presented herein expand on the currently reported structural information of cav-1. Peptides corresponding to the N-terminus of cav-1 displayed ~20%  *α*-helix, whereas the cav-1 C-terminal peptide unexpectedly showed 43%  *α*-helix, twice that of the N-terminus when peptides were analyzed by CD. This was surprising as the bioinformatics study predicted that the C-terminus would contain primarily random secondary structure. Hence, our data show that the cav-1 C-terminus contains helical structure, suggesting that it may not exclusively be random, and supports the consensus structural predictions of cav-1 aa 134–167 [52].

 CD analysis of NSP4-cav-1 peptide-peptide interactions revealed that there was no significant change in *α*-helix formation upon binding of NSP4_112-140_ and the N-Cav_19-40_ peptide. However, a structural change was noted upon interaction of NSP4_112-140_ with N-Cav_2-20_ and C-Cav_161-178_. Taken together with the binding assays, these results reveal that while the interaction between NSP4_112-140_ and N-Cav_19-40_ occurs with a strong affinity, binding does not require a conformational change. However, the secondary structure change observed when the NSP4_112-140_ and C-Cav_161-178_ peptides were mixed likely occurred to allow the two peptides to interact, albeit more weakly.

A statistically significant conformational change was observed when the NSP4_112-140_-Cav_68-80_ mixture was combined with model membranes. Since cav-1_68-80_ is located near the N-terminal membrane attachment domain (N-MAD; aa 82–101), as well as the transmembrane domain (aa 102–134) of cav-1, it was postulated that the observed conformational change was due to the interaction of Cav_68-80_ with the lipid vesicles. However, when Cav_68-80_ was mixed with SUVs in the absence of NSP4_112-140_, no change in secondary structure was observed. Extensive studies are needed to dissect how the SUVs alter the structure of the NSP4_112-140_ and Cav_68-80_ mixture.

 The interaction of NSP4 with both termini of cav-1 is unique, as most cav-1 binding proteins interact with the CSD [57, 58]. Since NSP4 (aa 112–140) interacted with the N-terminus of cav-1 more strongly than the C-terminus, it is possible that the interaction with the C-terminus is transitory. NSP4 activates signaling pathways that mobilize [Ca^2+^]_i_, requiring the protein to interact with membrane-associated signaling molecules [59, 60]. The weak binding between NSP4 and the C-terminus of cav-1 may serve to present and/or orient the viral protein for interactions with signaling molecules or lipids, such as the phosphoinositides. Once NSP4 is properly presented to signaling molecules within the membrane, the C-terminus of cav-1 may then quickly disassociate thus allowing NSP4 to carry out its biological function(s). Since the C-terminus of the cav-1 protein is closely associated with membranes and does not extend into the cytoplasm to the same extent as the N-terminus, it likely has less flexibility and/or accessibility for interactions with other proteins. This lack of freedom is another potential explanation for the weak binding observed between NSP4 and the C-terminus of cav-1.

It has been shown that NSP4 is released from RV-infected cells in different forms dependent on the cell type, virus strain, and MOI [36, 61, 62]. The differential binding of NSP4 to the N- and C-termini of cav-1 could also contribute to the presentation and transport of NSP4 across the PM and provide a key target to block NSP4 from exiting the cell. Many models can be envisioned to explain how NSP4 interacts with both cav-1 termini. Results of the fluorescence-binding assay showed that the N- and C-termini of cav-1 do not interact, so this possibility can be ruled out. It is likely that different molecules of NSP4 associate with the individual cav-1 termini.

Lastly, we demonstrated that an RV NSP4 peptide directly interacted with cholesterol and this interaction overlapped the amphipathic *α*-helix, the enterotoxic peptide, and cav-1 binding domains (aa 112–140), as well as a putative CRAC motif. The interaction between cholesterol and the NSP4 CRAC sequence has implications for the trafficking of NSP4 in RV-infected cells. Given that caveolae are involved in *de novo* cholesterol transport from the ER to PM caveolae [30, 39] and cav-1 assists in cholesterol transport [29, 30, 63, 64], contains a CRAC motif, and binds cholesterol in a 1 : 1 ratio, it is reasonable to propose that cav-1 and cholesterol function in NSP4 intracellular localization and transport [65, 66]. Indeed the interaction between NSP4 and cholesterol supports the hypothesis that NSP4 traffics via a cholesterol transport pathway in RV-infected cells. Likewise, NSP4 interacts with cav-1, has a proposed CRAC motif, traffics to the cell surface via a Golgi-bypassing, unconventional pathway [7, 34, 36, 67], and, as we show herein, interacts with cholesterol with a *K*_*d*_ of 8 ± 1 nM. We also showed that the NSP4-cholesterol interaction was specific to aa 112–140, as there was no binding to a C-terminal NSP4_150-175ΔAla_ peptide.

Based on these results using synthetic peptides, we hypothesize that NSP4 traffics with cholesterol and cav-1 from the ER to the PM, and to viroplasms via association to cholesterol. Additional studies are needed to verify this hypothesis, dissect NSP4 transport to the PM, and resolve viroplasm localization, all of which may constitute novel targets of reducing NSP4 effects.

## Figures and Tables

**Table 1:** 

Peptide name	Peptide sequence (N→C)
NSP4_112-140_ (aa 112–140)^a^	MIDKLTTREIEQVE**L**LKRI**Y**D**K**LTVQTTG
NSP4_150-175ΔAla_	QKNVRTLEEWESGKNPYEPREVTAM

N-Cav_2-20_ (aa 2–20)	SGGKYVDSEGHLYTVPIRE
N-Cav_19-40_ (aa 19–40)	REQGNIYKPNNKAMADELSEKQ
Cav_68-80_ (aa 68–80)	FEDVIAEPEGTHS
C-Cav_161-178_ (aa 161–178)	EAVGKIFSNVRINLQKEI

^a^Underlined and bold residues indicate the putative CRAC domain.

**Table 2:** Binding affinities (*K*_*d*_) for NSP4_112-140_ + caveolin-1 peptides in the absence and presence of SUV model membranes^a^.

Sample	*K*_*d*_ (nM) in absence of SUV	*K*_*d*_ (nM) in presence of SUV
Cy3-N-Cav_2-20_ + NSP4_112-140_	85 ± 6	26 ± 4
Cy3-N-Cav_19-40_ + NSP4_112-140_	40 ± 10	37 ± 7
Cy3-C-Cav_161-178 _+ NSP4_112-140_	217 ± 35	172 ± 22

^a^The *K*_*d*_  values were calculated as outlined in Materials and Methods and are presented as means ± SD,  *n* = 4.

**Table 3:** Percent *α*-helix^a^ of NSP4_112-140 _and Caveolin-1 Peptides in Aqueous Buffer, 50% TFE and in the Presence of 1 mM SUV.

Peptide	Aqueous Buffer	50% TFE	1 mM SUV
NSP4_112-140_	30.6 ± 2.4%	79.6 ± 4.6%	57.9 ± 1.6%
N-Cav_2-20_	18.9 ± 2.8%	16.5 ± 0.9%	18.5 ± 1.9%
Cav_19-40_	20.7 ± 0.6%	33.4 ± 0.5%	20.5 ± 2.7%
Cav_68-80_	23.4 ± 0.9%	24.4 ± 0.8%	21.4 ± 2.0%
C-Cav_161-178_	43.4 ± 3.8%	57.4 ± 4.4%	42.5 ± 10.6%

^a^Percent *α*-helix for each peptide was calculated as described in Materials and Methods. Data is presented as mean ± SD, *n* = 4.

**Table 4:** Observed and theoretical % helical content of NSP4_112-140_-caveolin-1 peptide-peptide interactions.

	N-Cav_2-20_		Cav_19-40_		Cav_68-80_		C-Cav_161-178_	
	Observed	Theoretical	Observed	Theoretical	Observed	Theoretical	Observed	Theoretical
Aqueous buffer	21.7 ± 0.8	23.6 ± 2.6	23.8 ± 1.2	24.4 ± 0.6	24.2 ± 0.3	23.9 ± 0.7	25.0 ± 1.7*	35.3 ± 1.6
50% TFE	46.3 ± 7.2	47.6 ± 1.8	58.7 ± 7.7	56.3 ± 2.3	45.9 ± 2.8	49.8 ± 1.8	44.2 ± 10.6*	67.8 ± 3.2
1 mM SUV	34.7 ± 6.0	37.7 ± 1.9	37.7 ± 3.6	39.0 ± 2.3	31.3 ± 0.2*	37.5 ± 1.9	51.9 ± 2.4	49.5 ± 6.2

NSP4_112-140_-Caveolin-1 Peptide-Peptide Interactions: Percent *α*-helical content of NSP4_112-140_ peptide + Caveolin-1 peptides, in aqueous buffer (10 mM potassium phosphate buffer), 50% trifluoroethanol (TFE) and in the presence of 1 mM SUV (55 : 35 : 10, POPC/cholesterol/DOPS). Observed values were compared with theoretical values, and those that were statistically significant by a Student's *t*-test (*P* < 0.05; denoted by an asterisk) were considered structurally altered, indicative of an interaction. Results are presented as means ± S.D. (*n* = 3 or 4).

**Table 5:** Treatment with cholesterol-altering drugs^a^.

Inhibitor	Action	Results
Fillipin	Cholesterol-binding; disrupts caveolae and caveolar membrane transport.	Disrupted NSP4 transport
Nystatin	Cholesterol-binding; disrupts caveolae and caveolar membrane transport.	Disrupted NSP4 transport
Chlorpromazine	Inhibits clathrin-dependent endocytosis and coated pit processes.	Normal NSP4 transport to the PM.
Nocodazole	Disrupts microtubule polymerization	Disrupted NSP4 transport

^a^Each inhibitor was reacted with RV-infected MDCK cells. The cells were washed, stained for NSP4 (anti-NSP4_150-175_) and a fluorescent secondary antibody (rabbit anti-IgG-FITC), and the intracellular location compared to untreated, infected cells. Chlorpromazine and nocodazole were included as controls.

## References

[1] Fischer TK, Viboud C, Parashar U (2007). Hospitalizations and deaths from diarrhea and rotavirus among children <5 years of age in the United States, 1993–2003. *Journal of Infectious Diseases*.

[2] Parashar UD, Alexander JP, Glass RI (2006). Prevention of rotavirus gastroenteritis among infants and children. Recommendations of the Advisory Committee on Immunization Practices (ACIP). *MMWR*.

[3] Ball JM, Tian P, Zeng CQY, Morris AP, Estes MK (1996). Age-dependent diarrhea induced by a rotaviral nonstructural glycoprotein. *Science*.

[4] Dong Y, Zeng CQY, Ball JM, Estes MK, Morris AP (1997). The rotavirus enterotoxin NSP4 mobilizes intracellular calcium in human intestinal cells by stimulating phospholipase C-mediated inositol 1,4,5-trisphosphate production. *Proceedings of the National Academy of Sciences of the United States of America*.

[5] Morris AP, Scott JK, Ball JM, Zeng CQ-Y, O'Neal WK, Estes MK (1999). NSP4 elicits age-dependent diarrhea and Ca^2+^-mediated I- influx into intestinal crypts of CF mice. *American Journal of Physiology*.

[6] Tian P, Estes MK, Hu Y, Ball JM, Zeng CQY, Schilling WP (1995). The rotavirus nonstructural glycoprotein NSP4 mobilizes Ca^2+^ from the endoplasmic reticulum. *Journal of Virology*.

[7] Bergmann CC, Maass D, Poruchynsky MS, Atkinson PH, Bellamy AR (1989). Topology of the non-structural rotavirus receptor glycoprotein NS28 in the rough endoplasmic reticulum. *The EMBO Journal*.

[8] Au KS, Mavoungou E, Estes MK (1993). A subviral particle binding domain on the rotavirus nonstructural glycoprotein NSP28. *Virology*.

[9] Boshuizen JA, Rossen JWA, Sitaram CK (2004). Rotavirus enterotoxin NSP4 binds to the extracellular matrix proteins laminin-*β*3 and fibronectin. *Journal of Virology*.

[10] O’Briek JA, Taylor JA, Bellamy AR (2000). Probing the structure of rotavirus NSP4: a short sequence at the extreme C terminus mediates binding to the inner capsid particle. *Journal of Virology*.

[11] Seo NS, Zeng CQY, Hyser JM (2008). Integrins *α*1*β*1 and *α*2*β*1 are receptors for the rotavirus enterotoxin. *Proceedings of the National Academy of Sciences of the United States of America*.

[12] Xu A, Bellamy AR, Taylor JA (2000). Immobilization of the early secretory pathway by a virus glycoprotein that binds to microtubules. *The EMBO Journal*.

[13] Bowman GD, Nodelman IM, Levy O (2000). Crystal structure of the oligomerization domain of NSP4 from rotavirus reveals a core metal-binding site. *Journal of Molecular Biology*.

[14] Ball JM, Parr RD, Schutt CE, Patton NA (2008). Genetic, structural and functional analyses of rotavirus NSP4. *Structure and Molecular Biology of Segmented Double-Standed RNA Viruses*.

[15] Taylor JA, O’Brien JA, Yeager M (1996). The cytoplasmic tail of NSP4, the endoplasmic reticulum-localized non-structural glycoprotein of rotavirus, contains distinct virus binding and coiled coil domains. *The EMBO Journal*.

[16] Liu L, Abramowitz J, Askari A, Allen JC (2004). Role of caveolae in ouabain-induced proliferation of cultured vascular smooth muscle cells of the synthetic phenotype. *American Journal of Physiology*.

[17] Vishwanathan SA, Thomas A, Brasseur R, Epand RF, Hunter E, Epand RM (2008). Hydrophobic substitutions in the first residue of the CRAC segment of the gp41 protein of HIV. *Biochemistry*.

[18] Murata M, Peränen J, Schreiner R, Wieland F, Kurzchalia TV, Simons K (1995). VIP21/caveolin is a cholesterol-binding protein. *Proceedings of the National Academy of Sciences of the United States of America*.

[19] Epand RM (2006). Cholesterol and the interaction of proteins with membrane domains. *Progress in Lipid Research*.

[20] Vincent N, Genin C, Malvoisin E (2002). Identification of a conserved domain of the HIV-1 transmembrane protein gp41 which interacts with cholesteryl groups. *Biochimica et Biophysica Acta*.

[21] Schroeder C, Heider H, Möncke-Buchner E, Lin TI (2005). The influenza virus ion channel and maturation cofactor M2 is a cholesterol-binding protein. *European Biophysics Journal*.

[22] Asano K, Asano A (1988). Binding of cholesterol and inhibitory peptide derivatives with the fusogenic hydrophobic sequence of F-glycoprotein of HVJ (Sendai virus): possible implication in the fusion reaction. *Biochemistry*.

[23] Parr RD, Storey SM, Mitchell DM (2006). The rotavirus enterotoxin NSP4 directly interacts with the caveolar structural protein caveolin-1. *Journal of Virology*.

[24] Mir KD, Parr RD, Schroeder F, Ball JM (2007). Rotavirus NSP4 interacts with both the amino- and carboxyl-termini of caveolin-1. *Virus Research*.

[25] Ball JM, Mitchell DM, Gibbons TF, Parr RD (2005). Rotavirus NSP4: a multifunctional viral enterotoxin. *Viral Immunology*.

[26] Isshiki M, Anderson RGW (1999). Calcium signal transduction from caveolae. *Cell Calcium*.

[27] Isshiki M, Anderson RGW (2003). Function of caveolae in Ca^2+^ entry and Ca^2+^ -dependent signal transduction. *Traffic*.

[28] Ikonen E (2008). Cellular cholesterol trafficking and compartmentalization. *Nature Reviews Molecular Cell Biology*.

[29] Ikonen E, Parton RG (2000). Caveolins and cellular cholesterol balance. *Traffic*.

[30] Smart EJ, Ying YS, Donzell WC, Anderson RGW (1996). A role for caveolin in transport of cholesterol from endoplasmic reticulum to plasma membrane. *Journal of Biological Chemistry*.

[31] Parton RG, Hanzal-Bayer M, Hancock JF (2006). Biogenesis of caveolae: a structural model for caveolin-induced domain formation. *Journal of Cell Science*.

[32] Huang H, Schroeder F, Estes MK, McPherson T, Ball JM (2004). Interaction(s) of rotavirus non-structural protein 4 (NSP4) C-terminal peptides with model membranes. *Biochemical Journal*.

[33] Huang H, Schroeder F, Zeng C, Estes MK, Schoer JK, Ball JM (2001). Membrane interactions of a novel viral enterotoxin: rotavirus nonstructural glycoprotein NSP4. *Biochemistry*.

[34] Storey SM, Gibbons TF, Williams CV, Parr RD, Schroeder F, Ball JM (2007). Full-length, glycosylated NSP4 is localized to plasma membrane caveolae by a novel raft isolation technique. *Journal of Virology*.

[35] Gibbons TF (2007). *Rotavirus NSP4 in extrareticular sites: support for its pathogenic role as an enterotoxin*.

[36] Gibbons TF, Storey SM, Williams CV, Schroeder M, Schroeder F, Ball JM (2011). 278–297 full-length, fully-glycosylated rotavirus NSP4 is exposed on the plasma membrane exofacial surface and released from rotavirus-infected cells. *Virology Journal*.

[37] Axén R, Porath J, Ernback S (1967). Chemical coupling of peptides and proteins to polysaccharides by means of cyanogen halides. *Nature*.

[38] Mitchell DMA, Ball JM (2005). Characterization of a spontaneously polarizing HT-29 cell line, HT-29/cl.f8. *In Vitro Cellular & Developmental Biology*.

[39] Conrad PA, Smart EJ, Ying YS, Anderson RGW, Bloom GS (1995). Caveolin cycles between plasma membrane caveolae and the Golgi complex by microtubule-dependent and microtubule-independent steps. *Journal of Cell Biology*.

[40] Orlandi PA, Fishman PH (1998). Filipin-dependent inhibition of cholera toxin: evidence for toxin internalization and activation through caveolae-like domains. *Journal of Cell Biology*.

[41] Silva L, Coutinho A, Fedorov A, Prieto M (2006). Competitive binding of cholesterol and ergosterol to the polyene antibiotic nystatin: a fluorescence study. *Biophysical Journal*.

[42] Schnitzer JE, Oh P, Pinney E, Allard J (1994). Filipin-sensitive caveolae-mediated transport in endothelium: reduced transcytosis, scavenger endocytosis, and capillary permeability of select macromolecules. *Journal of Cell Biology*.

[43] Chen YH, Yang JT, Chau KH (1974). Determination of the helix and *β* form of proteins in aqueous solution by circular dichroism. *Biochemistry*.

[44] Campbell L, Hollins AJ, Al-Eid A, Newman GR, Von Ruhland C, Gumbleton M (1999). Caveolin-1 expression and caveolae biogenesis during cell transdifferentiation in lung alveolar epithelial primary cultures. *Biochemical and Biophysical Research Communications*.

[45] Alberts AW (1988). Discovery, biochemistry and biology of lovastatin. *The American Journal of Cardiology*.

[46] Schroeder F, Frolov A, Schoer J, Freeman D, Chang TY (1998). Intracellular cholesterol binding proteins, cholesterol transport, and membrane domains. *Intracellular Cholesterol Trafficking*.

[47] Arias CF, Dector MA, Segovia L (2004). RNA silencing of rotavirus gene expression. *Virus Research*.

[48] Cheng G, Montero A, Gastaminza P (2008). A virocidal amphipathic *α*-helical peptide that inhibits hepatitis C virus infection in vitro. *Proceedings of the National Academy of Sciences of the United States of America*.

[49] Ball JM, Henry NL, Montelaro RC, Newman MJ (1994). A versatile synthetic peptide-based ELISA for identifying antibody epitopes. *Journal of Immunological Methods*.

[50] Brighty DW, Jassal SR (2001). The synthetic peptide P-197 inhibits human T-cell leukemia virus type 1 envelope-mediated syncytium formation by a mechanism that is independent of Hsc70. *Journal of Virology*.

[51] Fernandez I, Ying Y, Albanesi J, Anderson RGW (2002). Mechanism of caveolin filament assembly. *Proceedings of the National Academy of Sciences of the United States of America*.

[52] Spisni E, Tomasi V, Cestaro A, Tosatto SC (2005). Structural insignts into the function of human caveolin 1. *Biochemical and Biophysical Research Communications*.

[53] Sargiacomo M, Scherer PE, Tang Z (1995). Oligomeric structure of caveolin: implications for caveolae membrane organization. *Proceedings of the National Academy of Sciences of the United States of America*.

[54] Couet J, Li S, Okamoto T, Ikezu T, Lisanti MP (1997). Identification of peptide and protein ligands for the caveolin- scaffolding domain: implications for the interaction of caveolin with caveolae-associated proteins. *Journal of Biological Chemistry*.

[55] Anderson RGW (1998). The caveolae membrane system. *Annual Review of Biochemistry*.

[56] Anderson RGW, Jacobson K (2002). Cell biology: a role for lipid shells in targeting proteins to caveolae, rafts, and other lipid domains. *Science*.

[57] Li S, Couet J, Lisanti MP (1996). Src tyrosine kinases, G(*α*) subunits, and H-Ras share a common membrane-anchored scaffolding protein, caveolin: caveolin binding negatively regulates the auto-activation of Src tyrosine kinases. *Journal of Biological Chemistry*.

[58] Li S, Okamoto T, Chun M (1995). Evidence for a regulated interaction between heterotrimeric G proteins and caveolin. *Journal of Biological Chemistry*.

[59] Ruiz MC, Cohen J, Michelangeli F (2000). Role of Ca^2+^ in the replication and pathogenesis of rotavirus and other viral infections. *Cell Calcium*.

[60] Ruiz MC, Díaz Y, Peña F, Aristimuño OC, Chemello ME, Michelangeli F (2005). Ca^2+^ permeability of the plasma membrane induced by rotavirus infection in cultured cells is inhibited by tunicamycin and brefeldin A. *Virology*.

[61] Zhang M, Zeng CQY, Morris AP, Estes MK (2000). A functional NSP4 enterotoxin peptide secreted from rotavirus-infected cells. *Journal of Virology*.

[62] Bugarcic A, Taylor JA (2006). Rotavirus nonstructural glycoprotein NSP4 is secreted from the apical surfaces of polarized epithelial cells. *Journal of Virology*.

[63] Field FJ, Born E, Murthy S, Mathur SN (1998). Caveolin is present in intestinal cells: role in cholesterol trafficking?. *Journal of Lipid Research*.

[64] Robenek MJ, Schlattmann K, Zimmer K-P, Plenz G, Troyer D, Robenek H (2003). Cholesterol transporter caveolin-1 transits the lipid bilayer during intracellular cycling. *The FASEB Journal*.

[65] Martin S, Parton RG (2005). Caveolin, cholesterol, and lipid bodies. *Seminars in Cell and Developmental Biology*.

[66] Parton RG (1996). Caveolae and caveolins. *Current Opinion in Cell Biology*.

[67] Sapin C, Colard O, Delmas O (2002). Rafts promote assembly and atypical targeting of a nonenveloped virus, rotavirus, in Caco-2 cells. *Journal of Virology*.

